# Newly Diagnosed Idiopathic Thrombocytopenia Post COVID-19 Vaccine Administration

**DOI:** 10.7759/cureus.14853

**Published:** 2021-05-05

**Authors:** Precious O Idogun, Mindy C Ward, Yeshanew Teklie, Wilhelmine Wiese-Rometsch, Joel Baker

**Affiliations:** 1 Florida State University College of Medicine Internal Medicine Residency, Sarasota Memorial Hospital, Sarasota, USA

**Keywords:** covid 19, vaccination, itp, thrombocytopenia, adverse event

## Abstract

A few individuals are believed to have developed immune thrombocytopenia (ITP) following the administration of the coronavirus disease 2019 (COVID-19) vaccine. This phenomenon has been reported in a few case reports and also in some recent news articles. In this report, we discuss a case of a 54-year-old Caucasian female who presented to the emergency room with life-threatening thrombocytopenia in the setting of de novo ITP following COVID-19 vaccine administration. Due to the relapsing nature of ITP, it is unclear if the patient has achieved complete remission of the disease.

## Introduction

Primary immune thrombocytopenia (ITP) is a hematologic disorder characterized by isolated thrombocytopenia (platelet count of <100,000/uL) [1] without associated leukopenia or anemia [2]. Secondary ITP is defined as any form of ITP other than the primary kind. ITP is primarily a diagnosis of exclusion and newly diagnosed ITP usually refers to ITP diagnosed within the preceding three months. ITP is not a single disorder, but a syndrome in which thrombocytopenia may be primary or occur secondary to underlying infectious or immune processes. Vaccinations, especially the measles, mumps, and rubella (MMR) vaccine, have been implicated in the past as a possible trigger for its development [1]. Recently, a few case reports have shown a temporal relationship between receiving the coronavirus disease 2019 (COVID-19) vaccine and developing ITP of varying severity [3].

## Case presentation

A 54-year-old female presented with a three-week history of progressive and diffuse non-pruritic, painless, petechial rash on her lower extremities, chest, and abdomen. She reported increased mucosal bleeding and worsening ecchymosis.

**Figure 1:**
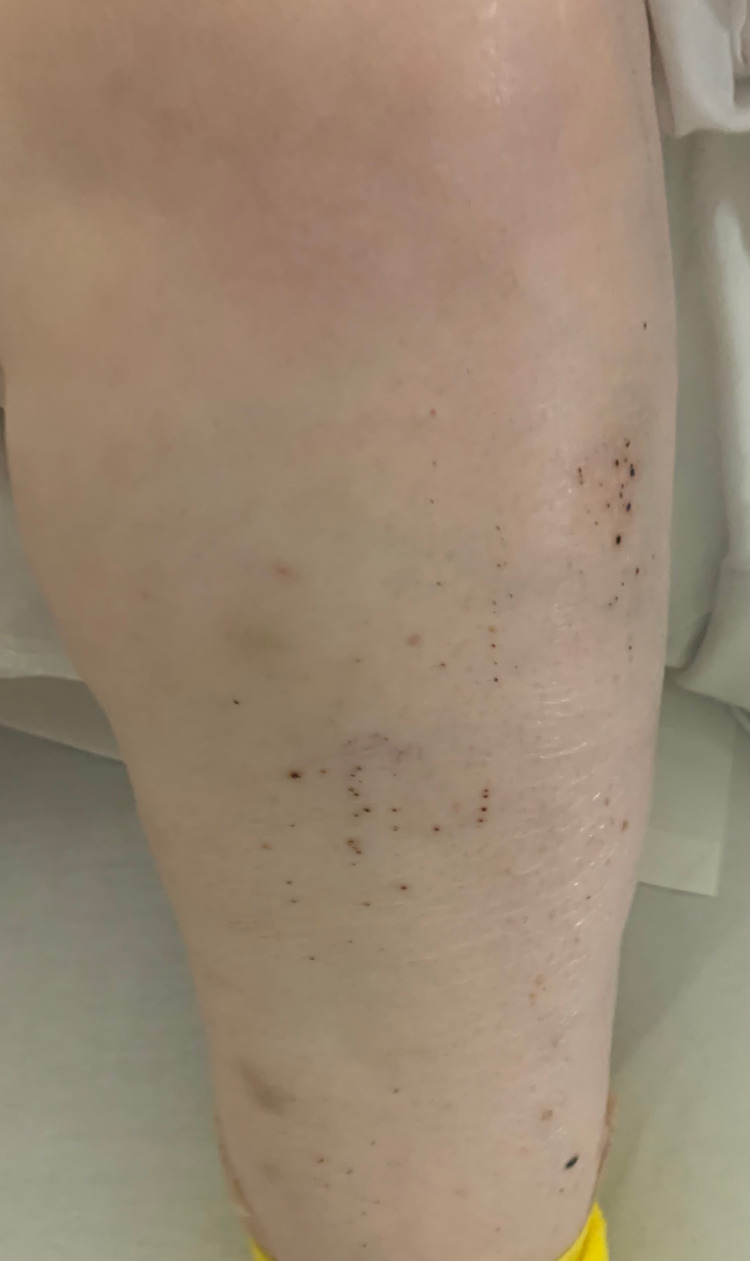
Petechiae located in the left lower extremity

**Figure 2:**
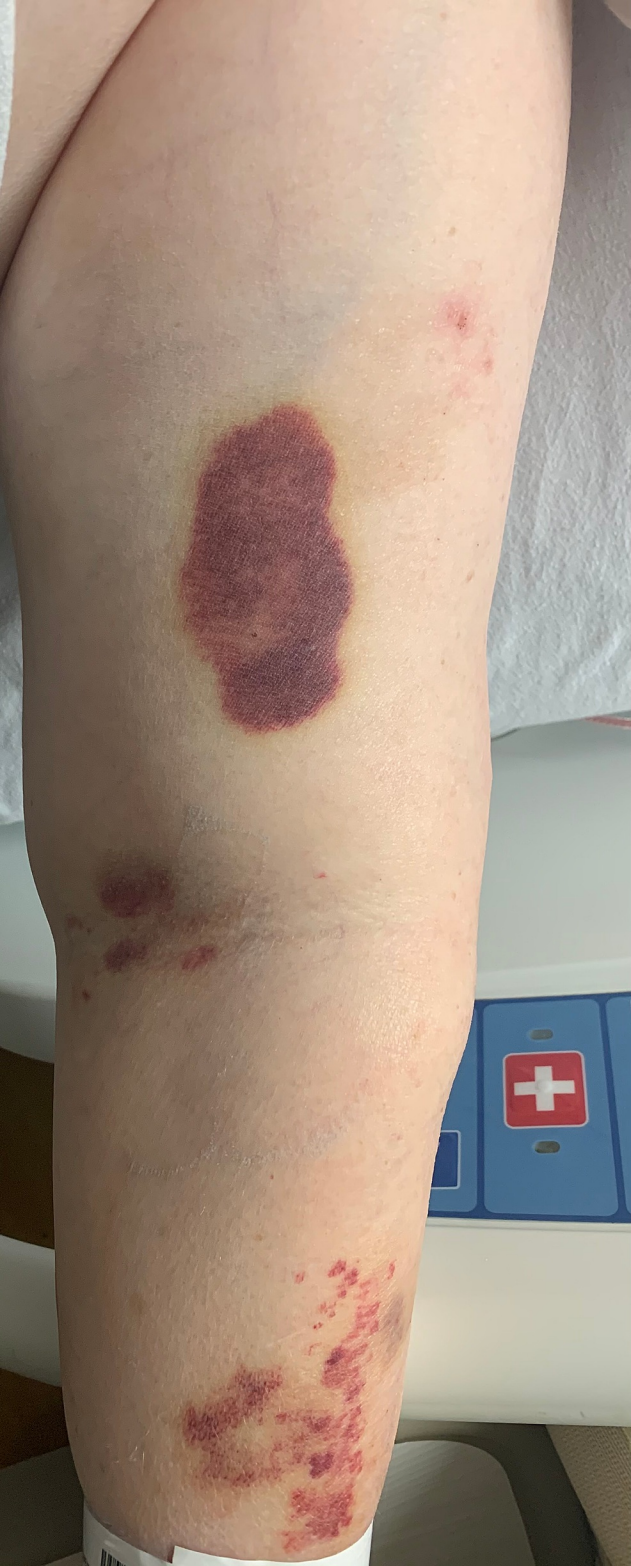
Ecchymosis of the left upper extremity

The patient had received the first dose of the Pfizer COVID-19 vaccine (Pfizer, New York City, NY) one week before the onset of the rash and had received the second dose five days prior to this presentation. Routine outpatient laboratory evaluation revealed a platelet count of zero, prompting hospital admission. A review of systems was otherwise negative as the patient denied pain, pruritus, fevers, or new lymphadenopathy. Physical examination was pertinent for elevated blood pressure at 184/95 mmHg and diffuse petechiae and ecchymoses, mostly in the bilateral upper and lower extremities (Figures 1, 2). There was no hepatosplenomegaly noted on the exam.

Her past medical history was significant for congenital epidermal dysplasia, hypertension, overactive bladder, mild cognitive impairment, chronic kidney disease, as well as anxiety. Her family history was insignificant except for colon cancer in her mother. The patient's home medications included amlodipine, lisinopril, pravastatin, baclofen, tizanidine, and sucralfate.

Initial laboratory findings showed a platelet count of 0/uL with an elevated D-dimer at 2.99 mg/L. Erythrocyte sedimentation rate (ESR) was elevated at 56 mm/hr, and the patient had mild transaminitis with alanine aminotransferase (ALT) of 56 U/L and aspartate aminotransferase (AST) of 45 U/L. Creatinine on presentation was 1.23 mg/dL with a baseline of 0.99 mg/dL.

**Figure 3:**
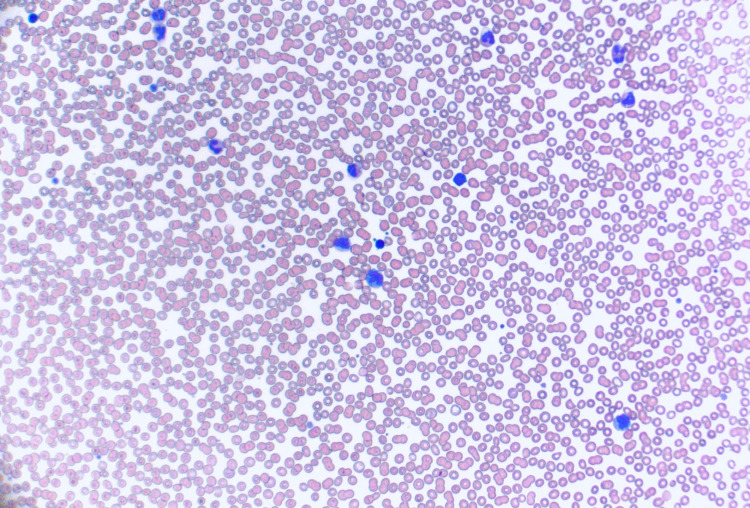
Initial peripheral smear demonstrating no visible platelets

CT of the head showed a right mastoid effusion with no acute intracranial hemorrhage. Serology was negative for hepatitis B virus, hepatitis C virus (HCV), Epstein-Barr virus, cytomegalovirus (CMV), or HIV infection. Additionally, severe acute respiratory syndrome coronavirus 2 (SARS-CoV-2) testing was negative. Bone marrow biopsy revealed a hypercellular bone marrow with megakaryocytic hyperplasia. Lactate dehydrogenase (LDH) and haptoglobin were within normal limits. Flow cytometry detected no overt immunophenotypic abnormalities. Initial peripheral smear confirmed no visible platelets (Figure 3).

Clinical course

Given that the patient's workup had been negative for secondary causes of thrombocytopenia, she was then started on dexamethasone as a treatment for presumptive ITP. Initial treatment consisted of three units of platelets and a 40-mg dose of IV dexamethasone with a good response. Her platelet count the following morning was 23,000/uL. An additional unit of platelets was given with further improvement in the count to 71k. Platelet counts continued to fluctuate as demonstrated in the graph below (Figure 3).

**Figure 4:**
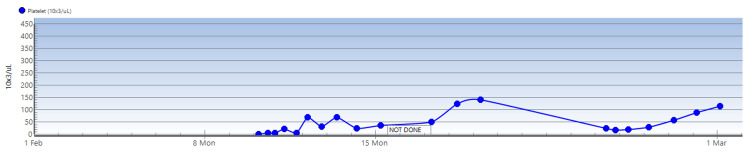
Graph demonstrating trends in platelet function during first and second hospitalizations

On the fifth day of hospitalization, platelet count remained low at 36,000/uL, and therapy was modified to include two doses of IV immunoglobulin (IVIG), followed by a two-day steroid taper. At the time of discharge, her platelet count was 135,000/uL. She was readmitted four days later for thrombocytopenia of 18k. She reported no additional complaints except for the emergence of new areas of ecchymosis on her forearms. At the second presentation, D-dimer and ESR/C-reactive protein (CRP) were again elevated. However, the direct Coombs test returned negative; fibrinogen, fibrinogen degradation products, haptoglobin, and LDH were within normal limits. Peripheral smear was without schistocytes, but immature platelet fraction was increased. Treatment consisted of four doses of dexamethasone with two days of IVIG treatment. During her second presentation, platelet transfusions were not administered. She was discharged four days later with a platelet count of 59k, and she has not been readmitted to date. Her laboratory test results over the course of her first and second presentation are summarized in Table 1.

**Table 1:** Table showing laboratory findings on admission and discharge WBC: white blood cells; RBC: red blood cells; MCV: mean corpuscular volume; MCH: mean corpuscular hemoglobin; MCHC: mean corpuscular hemoglobin concentration; RDW-SD: red cell distribution width-standard deviation; RDW-CV: red cell distribution width-coefficient of variation

Labs	Admission 1	Discharge 1	Admission 2	Discharge 2
WBC, x 10^9^/L	7.7	20.9	12.6	11.6
RBC, x 10^12^/L	3.72	4.17	3.78	3.74
Hemoglobin, g/dl	10.4	11.4	10.3	10.4
Hematocrit, %	33.0	35.4	33.9	32.4
MCV, fL	88.7	84.9	89.7	86.6
MCH, pg	28.0	27.3	27.2	27.8
MCHC, g/dL	31.5	32.2	30.4	32.1
RDW-SD, fL	58.4	54.0	56.7	55.7
RDW-CV, %	18.2	17.3	17.2	17.3
Platelet, k//uL	0	140	25	114

## Discussion

ITP is a complex autoimmune disease that is characterized by low platelet counts. The pathophysiology of ITP is uncertain; however, it is theorized that the acquired thrombocytopenia results from pathologic antiplatelet antibodies, impaired megakaryocytopoiesis, and T-cell-mediated destruction of platelets [4]. The annual incidence of ITP is approximately one to six per 100,000 adults while the prevalence in the United States is approximately 12 per 100,000 adults [2]. Most patients are asymptomatic at the time of diagnosis. In general, ITP most commonly occurs in healthy children and young adults within a few weeks following a nonspecific viral infection or other viruses such as HIV, HCV, Zika virus, CMV, or varicella-zoster virus (VZV). Autoimmune conditions such as systemic lupus erythematosus, antiphospholipid syndrome, and lymphoproliferative disorders have also been identified as secondary causes of ITP.

ITP has been associated with several types of vaccinations. Vaccine-related thrombocytopenia is considered to be of immune origin because antibodies can be detected on platelets in about 79% of cases [5]. Various reports have shown that all of the live, attenuated viruses in the measles, mumps, and rubella (MMR) vaccine can cause ITP whether administered alone or in combination [5]. MMR vaccine-related ITP usually occurs within six weeks of vaccination and the presentation is usually mild. Past studies have shown that the risk of developing ITP also increases after the administration of hepatitis A, varicella, and diphtheria-tetanus-pertussis vaccines in children and adolescents [5]. A few cases of ITP have been reported after influenza immunization in adults. However, studies examining the role of the influenza vaccine as a trigger of ITP have not proven definite causality [6,7].

The Vaccine Adverse Effect Reporting System (VAERS) is a passive, voluntary reporting system that collects reports of adverse events associated with vaccination. VAERS reports can be submitted voluntarily by anyone, including healthcare providers, patients, or family members [8]. A review of VAERS data has revealed 22 reports of thrombocytopenia and 13 reports of ITP following COVID-19 vaccine administration. However, underreporting is one of the main limitations of passive surveillance systems including VAERS, and it is possible that the true number of cases is far higher, especially considering that most cases of ITP are asymptomatic. The Vaccine Safety Datalink (VSD), on the other hand, conducts vaccine safety studies based on questions or concerns raised in the medical literature and from reports to VAERS [9]. As of now, the VSD database does not contain any confirmed cases of ITP following COVID-19 vaccine administration.

## Conclusions

Primary ITP is a hematologic disorder characterized by isolated thrombocytopenia. This case highlights the potential of the Pfizer vaccine to trigger de novo ITP. Given that 23.7 million people have received both doses of the COVID-19 vaccine so far, it would appear that the benefits of receiving the vaccine outweigh the risks of suffering from ITP. However, the COVID-19 vaccine has only been recently marketed and it is imperative that accurate surveillance must be planned in order to clearly evaluate for the incidence and emergence of all possible side effects related to it including ITP.

## References

[1] Kistangari G, McCrae KR (2013). Immune thrombocytopenia. Hematol Oncol Clin North Am.

[2] Donald M Arnold, MD MD, Adam Cuker MD (2021). Arnold DM, Cuker A: Immune thrombocytopenia (ITP) in adults: clinical manifestations and diagnosis. https://www.uptodate.com/contents/immune-thrombocytopenia-itp-in-adults-clinical-manifestations-and-diagnosis?search=itp&source=search_result&selectedTitle=1~150&usage_type=default&display_rank=1.

[3] Julian JA, Mathern DR, Fernando D (2021). Idiopathic thrombocytopenic purpura and the Moderna Covid-19 vaccine - a case report. Ann Emerg Med [Epub ahead of print].

[4] Lambert MP, Gernsheimer TB (2017). Clinical updates in adult immune thrombocytopenia. Blood.

[5] Cecinati V, Principi N, Brescia L, Giordano P, Esposito S (2013). Vaccine administration and the development of immune thrombocytopenic purpura in children. Hum Vaccin Immunother.

[6] Nagasaki J, Manabe M, Ido K (2016). Postinfluenza vaccination idiopathic thrombocytopenic purpura in three elderly patients. Case Rep Hematol.

[7] Hamiel U, Kventsel I, Youngster I (2016). Recurrent immune thrombocytopenia after influenza vaccination: a case report. Pediatrics.

[8] (2021). Vaccine Adverse Event Reporting System (VAERS). https://vaers.hhs.gov/.

[9] (2021). Vaccine Safety Datalink (VSD): Vaccine safety monitoring. https://www.cdc.gov/vaccinesafety/ensuringsafety/monitoring/vsd/index.html.

